# Integrating basic human values with forest ecosystem services: pathways to sustainable forest management

**DOI:** 10.3389/fpsyg.2024.1444775

**Published:** 2024-10-09

**Authors:** Darja Kobal Grum, Andrej Bončina

**Affiliations:** ^1^Department of Psychology, University of Ljubljana, Ljubljana, Slovenia; ^2^Department of Forestry and Renewable Forest Resources, Biotechnical Faculty, University of Ljubljana, Ljubljana, Slovenia

**Keywords:** environmental psychology, multi-objective forest planning, value hierarchy, public perceptions of forests, multifunctional forestry

## Abstract

The article explores the intricate relationship between basic human values and forest ecosystem services (FES). The study highlights the critical role that forests play in providing essential services for biodiversity, forest products, climate stabilization and human well-being, and emphasizes the need to understand and integrate human values into forest management and planning. Through a novel approach, this study explores how the concept of “forest” can elicit considerations of fundamental human values that diverge from conventional classifications and measurements of forest values. The study uses a comprehensive methodology, including surveys and content analysis, to uncover the hierarchical structure of human values associated with forests. This approach enables the identification of fundamental values that remain constant despite situational variations. The main results reveal a hierarchical structure of values, with Apollonian values being more prevalent than Dionysian ones. The study shows significant differences in the importance attributed to different FES, reflecting underlying value differences between residents. The study makes a novel contribution by systematically examining the links between human values and FES and proposing a profound and sustainable approach to forest management that takes into account the psychological dimensions of human-forest interactions. The study suggests that recognizing and incorporating the intrinsic human values into forest ecosystem service frameworks can improve sustainable forest management practices and ultimately foster a deeper connection between people and the forest environment.

## 1 Introduction

Forests play a critical role in providing a vast array of ecosystem services vital for biodiversity, climate stabilization, and human well-being. Despite increasing acknowledgment of these services’ significance, there remains a substantial disconnect in comprehending their correlation with basic human values. This paper endeavors to bridge this gap by delving into the integration of intrinsic human values within the realm of forest ecosystem services (FES). Our exploration commences with an examination of the prevailing discourse on FES and the values humans ascribe to forests. Finally, we discuss possible applications of our analyses for multi-valued forest planning and management.

Grasping both the functional essence of forests and the human values attributed to them is pivotal for crafting effective forest policies and management strategies. In recent years, the nexus between FES and forest-related values has emerged as a focal point of scholarly inquiry, aiming to enhance the understanding of human-forest interactions to refine forest management planning. The majority of existing studies (e.g., Bengston, 1994; Brown and Reed, 2000; Kellert, 1996; Reser and Bentrupperbäumer, 2005) concentrate on forest-specific values, which are inherently dynamic and thus pose challenges to consistent forest management planning. Our investigation seeks to identify enduring, foundational values that are impervious to situational fluctuations and remain relatively universal values. By anchoring our research on these immutable values, we aspire to facilitate profound and sustainable forest management strategies.

Recent studies in forest research (e.g., Grammatikopoulou and Vačkářová, 2021; Jones et al., 2016), predominantly utilize empirical methodologies to assess forests’ ecological, economic, and social contributions. While these approaches are valuable, they may not fully capture the nuanced, psychologically influenced dimensions that confer intrinsic value to forests from a human perspective. Although existing literature includes investigations into public perceptions and valuations of natural environments (e.g., Bakhtiari et al., 2014; Ordóñez Barona et al., 2021), there remains a significant gap in comprehensively exploring how individuals’ associations with forests indirectly reflect underlying human values.

This study seeks to pioneer research into this critical gap by examining how the concept of “forest” triggers semantic associations that reflect basic human values. Diverging from traditional methodologies that prioritize objective classifications and measurements, our investigation focuses on these semantic associations to explore the profound, subjective connections individuals form with forests. This approach enables us to systematically identify and organize a hierarchical structure of human values, providing deeper insights into human-nature relationships and emphasizing the broader values encapsulated in these interactions.

### 1.1 Basic human values

Broadly, values can be defined as concepts and beliefs that guide our lives (Howard, 1985; Rokeach, 1973). They refer to broad categories of subordinate objects and relationships that guide our interests and behavior (Musek, 2011, 2022, 2024). They are similarly defined as conceptions or beliefs about desirable end states or behaviors that transcend specific situations, guide, and direct the choice or evaluation of actions and phenomena, and are ranked according to their relative importance and valued as a guide in our lives (Sagiv and Schwartz, 2022; Schwartz, 2012). They transcend specific actions and situations, and they serve as standards or criteria, which means that guide the evaluation of actions. Values influence behavior when they are relevant in the context (hence likely to be activated) and important to the actor (Schwartz, 2012). Values are mental processes that are both cognitive and emotional. They combine cognitive representations such as concepts, goals, and beliefs with emotional attitudes that have positive or negative valence (Musek, 2015). Values are also like attitudes and beliefs in that they have cognitive, emotional, and behavioral parts. However, values are more abstract, but also more “central” than other constructs (Jones et al., 2016).

There are several different approaches and models of value theory that attempt to describe, classify, and measure values. Schwartz (2012) classified values into ten categories: stimulation, hedonism, achievement, power, security, conformity, tradition, benevolence, universalism, and self-direction. Stimulation, hedonism and partial security correspond to the hedonic value type, achievement and power to the potency type, conformity, tradition and benevolence to the moral type and universalism and self-direction to the fulfilment type. These values are divided into two dimensions: openness to change versus preservation and self-expression versus social orientation. This theory assumes that values are an expression of basic human needs and that they are in conflict or in harmony with each other. Recently, Schwartz and Cieciuch (2022) proposed a revised model of human values, which includes 19 value types. They can be divided into terminal and instrumental values. The former represent desirable end states (e.g., freedom, national equality, beauty), while the latter represent ideas about the behaviors that make it possible to achieve the desirable (e.g., honesty, courage).

**Figure 1:**
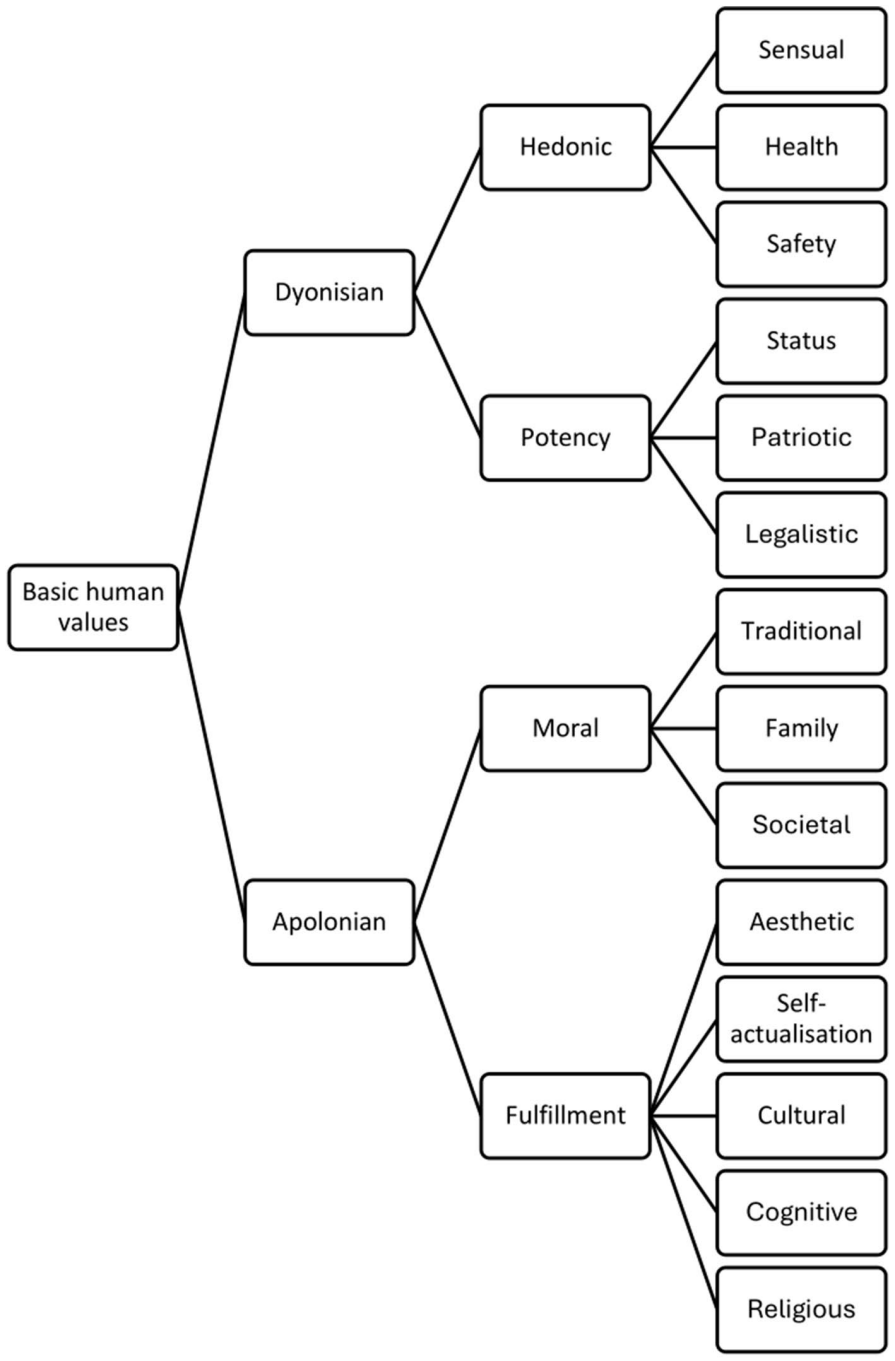
A complex theory of human values developed by Janek Musek [adopted from Musek (2000, 2015, 2022, 2024)].

Based on Rokeach and Schwarz’s classification of values Musek (2000, 2015, 2022, 2024) developed a complex theory of human values (Figure 1). He suggested several key assumptions:

1. Values are arranged in a hierarchical value space that comprises several levels depending on the generality or complexity of the value categories.
2. On representative levels of the structural hierarchy of values, the most important value categories or dimensions can be identified, including the most complex ones, which are few and relatively independent of each other.
3. There are developmental regularities in value orientation, so that adults at different stages of development place relatively higher importance on certain values and general value categories.
4. The most complex value categories are universal and independent of culture.
5. The less complex value categories are less universal and therefore more dependent on cultural influences.
6. There is a substantial relationship between values and important psychological, personal and demographic variables.
7. Values are linked to important life choices and behavioral patterns.
8. Important social transition processes are also reflected in changes in the values and value orientations of society.
9. Values play an important role in the integral functioning of the self.
10. Values are formed under the influence of a variety of factors, including biological, genetic, and evolutionary factors on the one hand and social, educational, and cultural factors on the other.

Musek (2000, 2015, 2022, 2024) places two categories of values at the top of the hierarchy, the first of which has more to do with sensual pleasure and achieving status. The second comprises moral and ethical values as well as values that strive for personal growth and self-realization. He named the two categories in reference to the ancient legend of Dionysus and Apollo, according to which the former embodied wildness and intemperance and the latter harmony and serenity: the Dionysian and Apollonian values (Figure 1). The first group combines the values of pleasure and achievement and includes the values of safety, health and patriotism. The second group includes moral and fulfilment values. Each of the broadest categories is subdivided into two broad categories or value types (four value types in total). Thus, Dionysian values are divided into the hedonic value type (values of pleasure and material goods, safety, and health) and the potency value type (values of achievement, accomplishment, social strength and, at least in part, patriotic values). Apollonian values are divided into moral values (traditional, social, and democratic or societal values) and fulfilling or humanistic values (cultural, cognitive, actualizing, esthetic, and religious values). Moral values are thus associated with responsibility and duty, while fulfilling values are associated with the fulfillment of life’s purpose and meaning.

Each of the four broad categories could be further subdivided to obtain the middle value categories: sensual values (pleasure, fun, exciting life), health values (health), safety values (security, peace of mind), status values (power, prestige, fame, money), patriotic values (love of country, national pride), legalistic values (order, respect for the law), traditional values (honesty, kindness, hard work), social and family values (family happiness, understanding for the partner, love, love for children), social and democratic values (equality, national equality, peace, style, justice), cultural values (culture, art, creativity), esthetic values (beauty, nature), actualizing values (knowledge, progress, self-improvement), cognitive values (truth, wisdom) and religious or spiritual values. Understanding these values is crucial when exploring human interactions with natural environments, such as forests, which are not only resources for physical needs but also for psychological fulfillment.

### 1.2 Forest ecosystem services and multifunctional forestry

In Central European (CE) forestry, the concept of multi-objective forest management, usually referred to as “multifunctional forestry” had been developed in the second half of the 20th century. It is oriented to provide multiple FES, traditionally called “forest functions” (Ger.: Waldfunktionenen) (Bončina, 2011; Bončina et al., 2019; Borrass et al., 2016; Dieterich, 1953). They have been integrated in the forestry legislation of CE countries and as policy framework for sustainable forest management also at the European level (Forest Europe, 2015; MCPFE, 2003). These FES are usually classified into three main groups, i.e., productive (economic), protective (environmental) and social (recreation, well-being). Considering the general classification scheme of ecosystem services (Maes et al., 2012) the first group fits mainly to provisioning, the second to the regulating, and the third one to the cultural group of ecosystem services. FES are conditioned by the societal demands towards forests; they indicate the relevancy of forests for society. The assessing, mapping and prioritizing of FES is a crucial part of multiple-objective forest planning. Productive FES are related for instances to timber production, income, game management, non-timber products; environmental to protection against natural hazards, regulation of water regime, biodiversity conservation, while social FES include well-being, recreational, aesthetic, educational aspects of forest use. These FES, integrated into the forestry legislation of CE countries and the European policy framework for sustainable forest management, demonstrate a formal recognition of the multifaceted roles forests play, beyond timber production to include protection and social services.

The concepts of FES and the concept of social values towards forests are interrelated, in some cases they may overlap (e.g., Koch and Kennedy, 1991). However, in the current concept of multi-objective forest management the main focus is still given to the provision of FES, and much less attention is paid to psychological and cultural human values attached to forests. Through a detailed analysis of public perceptions and values associated with forests, this study contributes to the evolving discourse on multi-objective forest management by integrating psychological and cultural dimensions into forest ecosystem service frameworks.

### 1.3 Rationale of the study

The primary goal addresses the crucial issue arising from the lack of understanding of how fundamental human values influence individuals’ connections with forests. This central concern includes several intertwined challenges: the difficulty of recognizing and comprehending these values and their prevalence; the ambiguity in categorizing these values into a coherent hierarchy that corresponds with established theoretical models; the obstacle of incorporating these intrinsic human values into the concept of FES; and the inadequate representation of these values in current frameworks for prioritizing FES. This comprehensive problem statement underscores the necessity for detailed exploration and innovative methods to close the gap between quantitative ecosystem assessments and qualitative value-based evaluations. Such efforts are essential to ensure that forest management and conservation policies are in harmony with societal values and expectations.

Furthermore, this research holds national significance for the maintenance of forest ecosystem services, as it incorporates a representative sample of forest users. This inclusion is pivotal for ensuring that the findings and subsequent strategies reflect the diverse interactions and values of the population regarding forest environments.

By pursuing these objectives, our study aims to deepen the understanding of the relationship between basic human values and forest management. The final objective is to devise more inclusive and effective strategies that resonate with societal values and promote sustainability, thereby contributing to maintain and enhance forest welfare services.

Based on the above, we put forward two hypotheses:

H1: There is a hierarchical structure of basic human values associated with forests, which can be systematically identified and organized based on individuals’ semantic associations with the concept of forest. These semantic associations indirectly capture underlying perceptions and attitudes, allowing for a systematic organization that reflects how people value forests.

H2: The importance attributed to different FES by participants varies significantly, reflecting underlying differences in their values of forests. This hypothesis explicitly connects the importance of FES with the variability in values and perceptions among residents, which can be explored by statistical analyses.

Both hypotheses are framed to reflect the anticipated analysis and findings. These refinements ensure that the hypotheses are not only testable but also closely aligned with the objectives of our research, providing a clear direction for our methodology and analysis.

## 2 Methods

### 2.1 Study area

**Figure 2:**
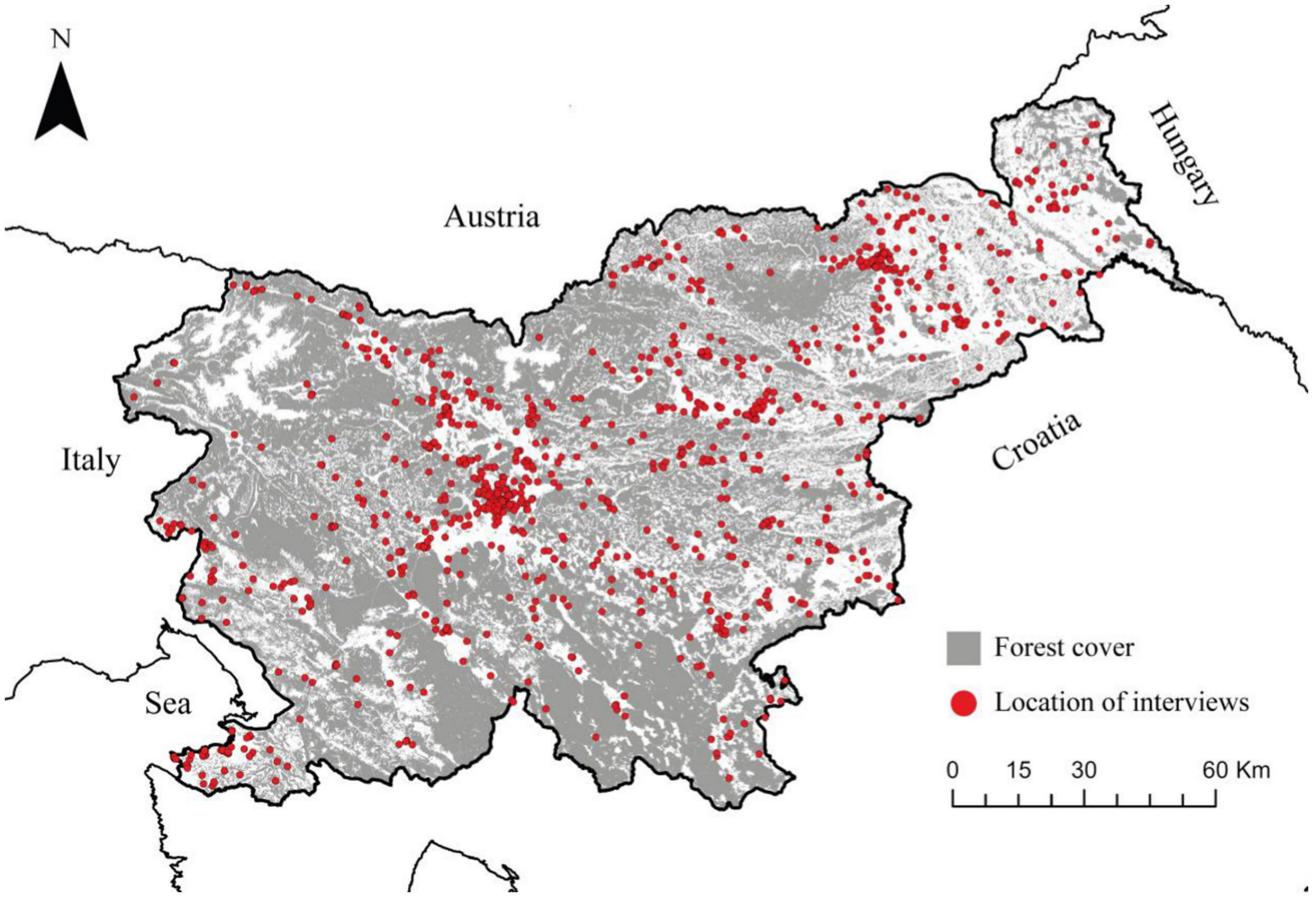
Forest cover of Slovenia (grey) with locations of respondents’ residences (red).

Slovenia is an example of Central European country with high percentage of forest cover (58%), practicing ecological forestry for more than 70 years. A clear-cutting system has been forbidden to apply since late nineteen-forties. Uneven-aged forestry is predominantly practiced, based on natural regeneration and mimicking natural stand development. Each tree for harvesting should be marked by the staff of Slovenia Forest Service. All forests should be managed according to the principles of sustainable, multi-objective and close-to-nature forest management. For the residents the forests have high societal values (Simončič et al., 2013). People have the right of free by free access to all forests, gathering of forest fruits and mushrooms are allowed in whole forest land, but limited to a certain amount. Strategic and operational forest management plans are prepared by Forest Service for the entire forest area. 80% of forests are privately owned, characterized by small scale private forest ownership. Figure 2 shows the forest cover of Slovenia with locations of respondents’ residences.

### 2.2 Participants

To get insight into perceptions of citizens towards forests a survey of adult citizens was conducted by computer aided telephone interviews with cell telephone and line telephone holders (70 and 30%, respectively) to avoid overrepresentation of the older population when sampling only among the latter. Citizens were sampled randomly in 12 statistical regions to provide the representative sample. Similar to other surveys on forest in Slovenia (e.g., Ficko and Bončina, 2019) the target number of respondents was 1,000 to achieve a suitable sampling error. Data were collected from a total of 1,158 residents. Women present 50.9% of all respondents, 27.0% of all respondents were forest owners, the average age of respondents was 54.2 years, most of them (57.6%) finished high school.

### 2.3 Materials and procedure

The questionnaire encompasses 19 questions, mainly of close-ended questions with 5-level ordinal Likert scale, few questions were dichotomous, only one question was open-ended. Two of questions were crucial for this study.

In the first question, the participants were given the following instruction: “People have different relationships with the ‘forest’. What are your associations when you hear the term forest? Please name up to three terms.” Based on this instruction, they listed one to three associations with the word forest. Respondents were asked to express their value perceptions about forests in their own terms. This seems to be more appropriate than using predetermined answers. Therefore, similarly to the procedure of Ordóñez Barona et al. (2021) we analyzed the original verbatim responses. We assigned a verbatim response to codes, which were then assigned to one of the psychological categories.

The second set of questions was about primary forest ecosystem services: “Importance of forests is quite different for people. How important for you personally are the following FES: recreation (x_1_), timber (x_2_), nature conservation (x_3_), protection against natural hazards (x_4_), aesthetic ecosystem service (x_5_), hunting (x_6_), gathering (x_7_), education and research (x_8_), climate regulation (x_9_). The respondents express their opinion by 5-level Likert scale (1, not important at all, …, 5, highly important).

### 2.4 Analyses of data

The data analysis process was designed to systematically test two hypotheses put forward in this study. To test Hypothesis 1, we employed content analysis to identify a hierarchical structure of basic human values associated with forests This approach allowed us to categorize semantic associations and organize them into a structured value hierarchy. To test Hypothesis 2, we utilized discriminant analysis to examine the variability in the importance attributed to different FES by participants, and how these variations reflect underlying differences in forest values.

#### 2.4.1 Content analysis of the responses regarding the semantic associations with the word “forest”

First, we analyzed frequency of initial verbatim responses. The row data of residents’ response on forests were visualized by using “worldcloud” package in R v. 4.1 (R Core Team, 2021). Row data were coded into value categories for which the frequency analyses were conducted. The respondents’ answers to the first question were sorted according to value categories on the basis of a content analysis. We used hierarchical system of value categories according to Musek’s comprehensive theoretical model of values (Musek, 2011, 2015, 2022, 2024). Based on the content analysis method, we conceptually categorized the individual values and divided them into hierarchically superordinate groups. A few associations directly described the forest (e.g., animal, leaves, plants) could not be assigned to any psychological category.

The purpose of this analysis was to confirm Hypothesis 1 by establishing whether a systematic and hierarchical structure of values could indeed be identified based on semantic associations with forests.

**Figure 3:**
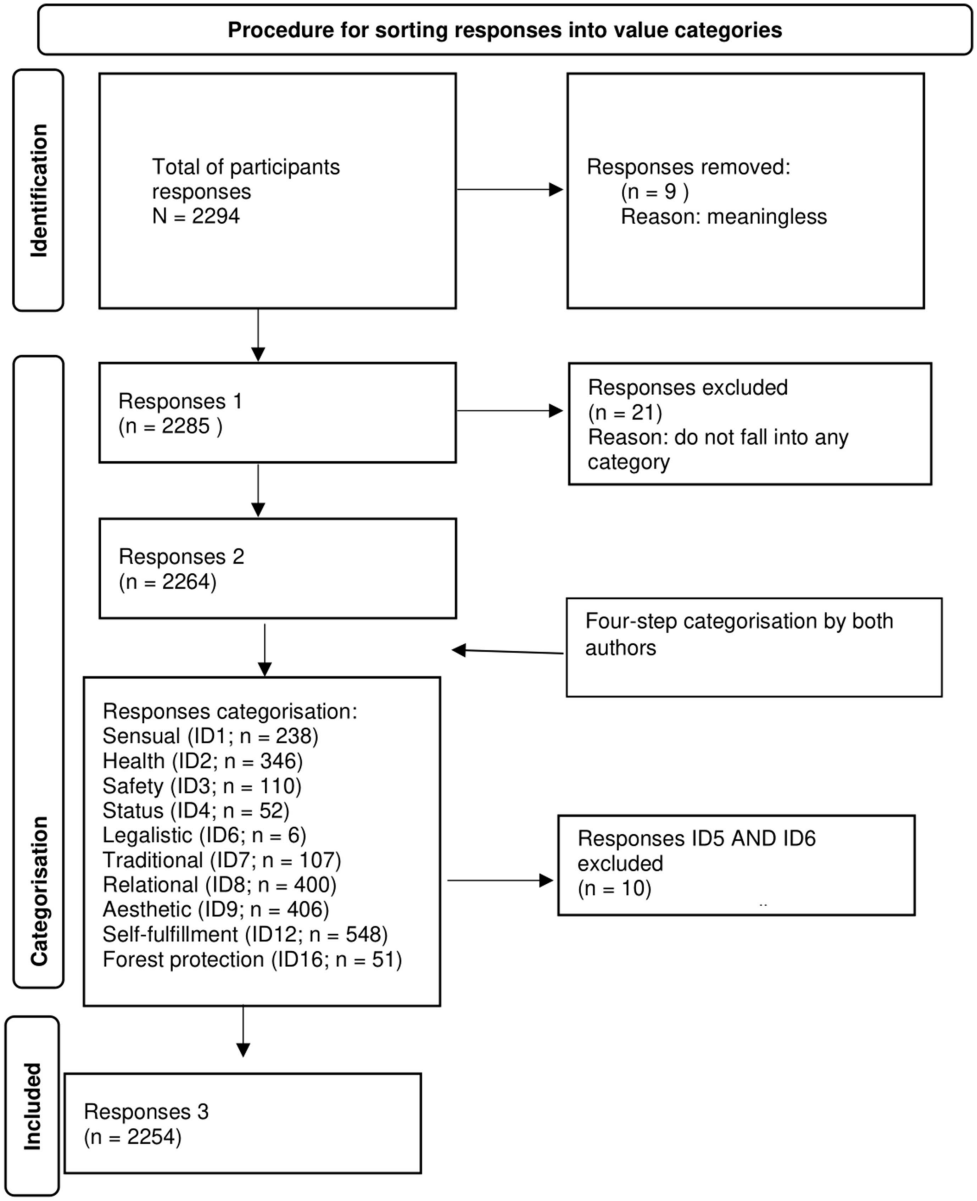
Sorting the responses to the word “forest” according to value categories (ID).

Figure 3 shows the ranking of responses according to each value category. There were a total of 2,294 responses, whether duplicated or not. Of the responses, we first excluded those that in our opinion did not relate to any value (e.g., do not know, nothing). There were 8 of these responses, leaving 2,287 responses. These responses were sorted into value categories in four steps: In the first step, the two authors sorted the responses into the categories together; in the second step, the first author completed the sorting; in the third step, the two authors jointly reviewed and completed the sorting together; and in the fourth and final step, the first author reviewed and sorted the responses again. This completed the ranking.

**Figure 4:**
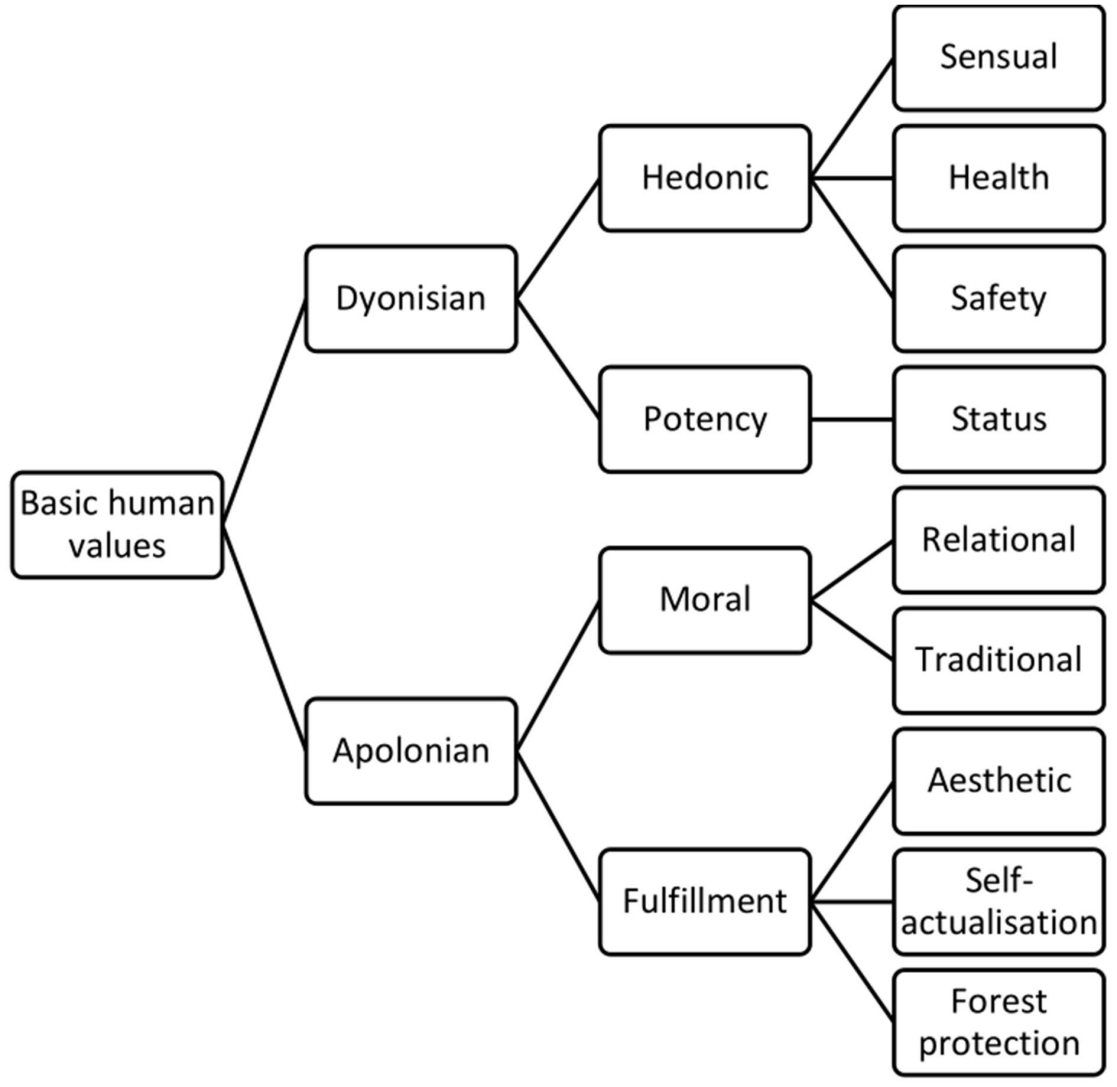
Conceptual model of the classification of values according to the qualitative content and quantitative frequency distribution of response to the word “forest”.

Based on our analysis, we have developed a conceptual model of the value hierarchy based on Musek’s model, but the participants’ responses (associations) have led us to a revised or modified form of the model. Due to the small number of associations, some of the value categories (family, social and cultural) were combined into the newly created category the “relational values,” which fits into the Apollonian value category. The classification of the basic value categories after the analysis is shown in Table 1 and Figure 4 as follows:

- the hedonic values’ category was retained: sensuality (ID 1), health (ID 2) and safety (ID 3);
- we have also retained the category of potency values and, at a lower level, only the basic category of status values (ID 4), as the other two categories cover too few associations for further analysis;
- we also kept the category of moral values, but at a lower level we kept two subcategories—traditional values (ID 7) and created a new category of relatedness (ID 8);
- we kept the category of fulfilment, but at a lower level we kept aesthetics (ID 11), self-fulfillment (ID 12), in which we combined the values from Musek’s model related to self-actualization, religion, spirituality and cognitive values, and we added a new subcategory of forest protection (ID 16).

**Table 1:** Conceptual model of the classification of values according to the qualitative content and quantitative frequency distribution of responses to the word “forest” with illustration how codes were defined and assigned to the psychological categories of forest values.

ID	Basic categories of forest values	Abbr.	Examples of responses	Cluster of categories	General clusters
1	Sensual	Sens	Leisure, touch, pleasure	Hedonic	Dionys
2	Health	Heal	Fresh air, health, recreation	Hedonic	Dionys
3	Safety	Safe	Safety, rest, shelter	Hedonic	Dionys
4	Status	Stat	Cashier, wealth, bank	Potency	Dionys
7	Traditional	Trad	Woodwork, mushroom picking, collecting firewood	Moral	Apollon
8	Relational	Rela	Socializing, habitat, attachment	Moral	Apollon
11	Aesthetic	Aest	Nature, greenery, beauty	Fulfillment	Apollon
12	Self-fulfillment	Self	Peace, personal satisfaction, relaxation	Fulfillment	Apollon
16	Forest protection	Prot	Cleanliness, forest protection	Fulfillment	Apollon

We understand that some values, such as “peace,” “personal satisfaction,” and “relaxation,” may intuitively seem more related to Dionysian values like “safety,” “rest,” “shelter,” “leisure,” “touch,” and “pleasure.” However, according to Musek’s model, our categorization is based on the distinction between values associated with personal growth and fulfillment (Apollonian) and those related to sensory pleasure and immediate gratification (Dionysian). In this context, “peace,” “personal satisfaction,” and “relaxation” were categorized under Apollonian values because they align more closely with the concept of self-fulfillment and long-term well-being, which are key aspects of the Apollonian category. This theoretical framework guided our analysis and helped us to systematically organize the values according to the underlying psychological constructs. We acknowledge that different theoretical perspectives might categorize these values differently, but we have adhered to Musek’s distinctions (Musek, 2015, 2022, 2024) to maintain consistency in our analysis.

#### 2.4.2 Discriminant analysis

To test Hypothesis 2, we employed discriminant analysis to explore how different value perceptions influence the prioritization of ecosystem services among respondents. This analysis aimed to determine whether the differences in the importance of FES could be linked to the value categories identified in Hypothesis 1.

We expected that respondents with different perception value prioritize ecosystem services in different way. We were interested if questions related to ecosystem services (x_1_… x_9_) can differentiate value categories. Therefore, stepwise linear discriminant analyses (SLDA) were applied to find a linear combination of the variables x_i_ that characterize forest value categories. The forward method was used to select the variables with the highest discriminant power for differentiating the value categories. The predictors were selected by using the *F*-statistics (minimum *p*-value <0.05). An analysis of variance (ANOVA) was then conducted to validate the discriminant model and ensure that the selected variables indeed reflected significant differences across value categories. Additionally, a least significant difference test (LSD) at a significance level of *p* < 0.05 was performed for the variables selected by the SLDA model and additionally for all variables x_i_ to identify pairs of forest value categories with significant differences in individual predictors. Statistical analyses were performed in R v. 4.1 (R Core Team, 2021).

## 3 Results and discussion

This section presents the findings from both the content analysis and discriminant analysis, linking each result directly to the hypotheses they were intended to test.

**Figure 5:**
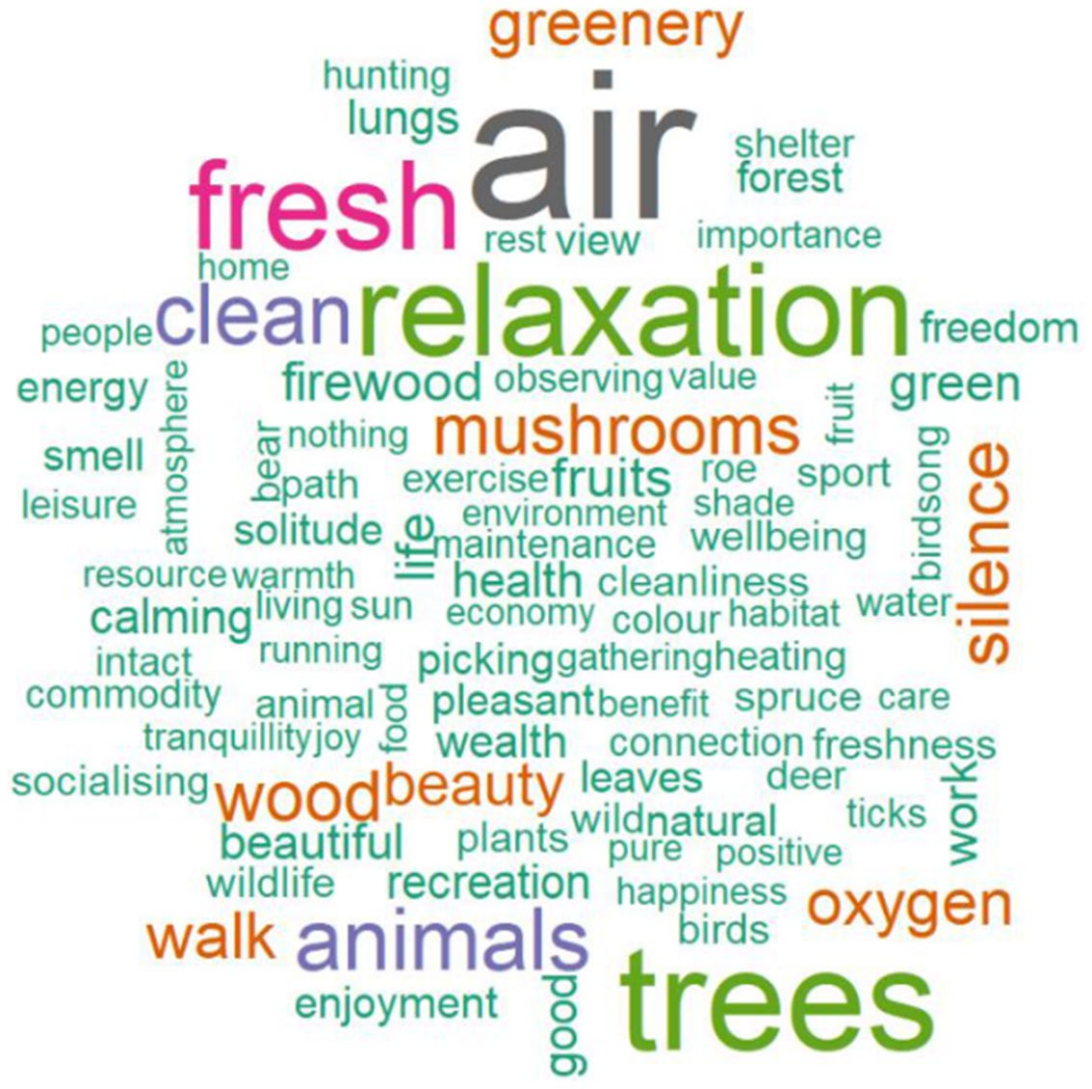
Visualization of associations related to the “forest” using the “wordcloud” package in R v. 4.1 (R Core Team, 2021).

Figure 5 shows a word cloud of associations to the word “forest.” Verbatims that were used more frequently are shown in larger letters. It turns out that the most frequently recorded word was air and nature, followed by relaxation and trees.

Content analysis was used to classify participants’ responses into 11 value categories, 9 of which were selected for further analysis due to the small number of responses (Figure 3; Table 1). The largest percentage of responses, namely 24.3%, was assigned to the self-actualization category, followed by aesthetics (18.0%), relational (17.8%), health (15.4%), sensuality (10.7%), safety (4.9%), tradition (4.8%), status (2.3%) and forest protection (2.3%). Patriotic (0.2%) and legalistic (0.3%) were underrepresented and were therefore not included in the further analysis.

If we place the above value categories one hierarchical level higher, as predicted by Musek’s theoretical model, we find that the following are expressed at this level: hedonic (30.8%) and potency (2.8%), which form the Dionysian category (33.5%). In the case of Apollonian values, however, it turns out that three separate value categories are formed, rather than the two that Musek’s model of value hierarchy and structure predicts. These are: traditional (4.9%), relational (17.8%) and fulfilling (44.6%). This means that the participants in our study express significantly more Apollonian values (67.2%) than Dionysian ones (44.6%) (Figure 4).

These results confirm Hypothesis 1, demonstrating that a hierarchical structure of values can indeed be systematically identified based on semantic associations with forests. The hierarchical model derived from the analysis aligns closely with the expectations laid out in the hypothesis.

Five of nine variables were selected by the stepwise linear discriminant analysis (SLDA) to differentiating the human categories (Table 2; Appendix 1). The recreation (x_1_) was the variable that contributed most to the separation of categories, followed by the timber (x_2_), climate regulation (x_9_), hunting (x_6_) and protection against natural hazards (x_4_).

**Table 2:** Results of stepwise discriminant analysis (Wilks’ Lambda: 0.9056; *p* < 0.0000).

Variables	Wilks’ lambda	F statistics	*p*-value	F to remove
x_1_	0.9683	8.9031	0.0000	8.9031
x_2_	0.9524	6.7245	0.0000	4.5667
x_9_	0.9400	5.6778	0.0000	3.5947
x_6_	0.9300	4.9811	0.0000	2.8976
x_4_	0.9225	4.4281	0.0000	2.2227

x_1_, recreation; x_2_, timber; x_9_, climate regulation; x_6_, hunting; x_4_, protection against natural hazards.

**Figure 6:**
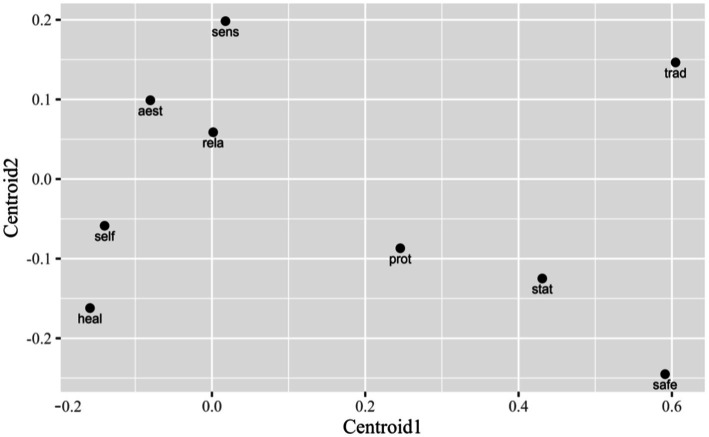
Plot of centroids for human values using discriminant functions 1 and 2.

Figure 6 shows (dis)similarities between human values in regard to the discriminant analysis, while Table 3 shows significant differences inside of pairs of value categories. For instance, sensual, aesthetic, self-fulfillment and relational, are quite close (similar) considering the relation to ecosystem services, no significant differences exist between them (se pravi izppustimo variable, ki so navedene v Table 4) differences in four variables (x_1_, x_2_, x_4_, x_6_) exist between them. In the opposite, there are quite big distances and significant differences between safety and traditional, and other human value categories (Figure 6; Table 3).

**Table 3:** Significant differences (*p* < 0.05) between basic value categories based on SLDA.

Human values	Safety	Status	Traditional	Relational	Aesthetic	Self-fulfillment
Sensual	***		***			
Health	***	**	***			
Safety				***	***	***
Status					*	**
Traditional				***	***	***

Only pairs with significant differences are shown. ***p < 0.001; **p < 0.01; *p <0.05.

The discriminant analysis supports Hypothesis 2 by showing significant differences in the importance attributed to different FES based on underlying value differences among participants. The analysis reveals that value categories can indeed predict the prioritization of ecosystem services, thereby confirming the hypothesis.

Significant differences between categories exist in all nine variables (ANOVA; *p* < 0.05); Post-hoc analyses (Table 4) showed that the most significant differences inside of pairs of human values were found for the recreation (x_1_; 15 pairs), less for timber (x_2_; 13 pairs), climate (x_9_; 13 pairs), hunting (x_6_; 9 pairs) and protection (x_4_; 8 pairs). Significant differences between values categories exist also in variables not included in the SLDA: nature conservation (x_3_; 7 pairs), aesthetic (x_5_; 5 pairs), gathering (x_7_; 3 pairs), education and research (x_8_; 6 pairs).

**Table 4:** Significant differences between basic value categories in the variables indicating importance of ecosystem services.

Human values	Health	Safety	Status	Traditional	Relational	Aesthetic	Self-actualization	Forest protection
Sensual	x_3_, x_5_, **x**_**9**_	**x**_**1**_, **x**_**2**_	**x**_**1**_, **x**_**2**_	**x**_**1**_, **x**_**2**_	**x**_**6**_, x_7_	x_3_, x_8_, **x**_**9**_	x_3_, **x**_**6**_, x_8_, **x**_**9**_	x_3_, x_8_, **x**_**9**_
Health		**x**_**1**_, **x**_**2**_, **x**_**4**_, x_5_, **x**_**9**_	**x**_**1**_, x_4_, **x**_**9**_	**x**_**1**_, **x**_**4**_, x_5_, **x**_**6**_, **x**_**9**_	**x**_**1**_, **x**_**2**_, x_3_, **x**_**4**_, x_5_, x_7_, **x**_**9**_	x_5_, x_7_, **x**_**9**_	**x**_**4**_, x_5_, **x**_**9**_	
Safety					**x**_**1**_, **x**_**2**_	**x**_**1**_, **x**_**2**_, **x**_**4**_	**x**_**1**_, **x**_**2**_, **x**_**6**_	**x**_**2**_
Status					**x**_**2**_		**x**_**1**_	
Traditional					**x**_**1**_, **x**_**2**_	**x**_**2**_, **x**_**4**_, **x**_**6**_, **x**_**9**_	**x**_**1**_, **x**_**2**_, **x**_**6**_, x_8_, **x**_**9**_	**x**_**9**_
Relational						x_3_, x_4_, x_8_	**x**_**1**_, x_3_, x_8_	x_3_, **x**_**6**_
Aesthetic							**x**_**6**_	
Self-actualization								**x**_**1**_, **x**_**6**_

Variables selected by the SLDA are in bold. x_1_, recreation; x_2_, timber; x_3_, nature conservation; x_4_, protection against natural hazards; x_5_, aesthetic value; x_6_, hunting; x_7_, gathering; x_8_, education and research; x_9_, climate regulation.

The ANOVA results further confirm the findings from the discriminant analysis, providing robust support for Hypothesis 2.

The discriminant analysis supports Hypothesis 2, indicating significant differences in the importance attributed to different FES based on underlying value differences among participants. This finding is consistent with previous research indicating that individual values significantly influence environmental perceptions and priorities (Gifford and Nilsson, 2014; Schaefer et al., 2020). This variability reflects the complex and multifaceted nature of human-forest interactions. For instance, health and forest protection values were strongly associated with climate regulation and protection against natural hazards (Appendix 1), highlighting a collective awareness of climate change and the necessity for forest conservation (Watson et al., 2018; Weiss, 2000). This is also highlighted by Unterberger and Olschewski (2021), who emphasize the significant role of forests in natural hazard protection and the willingness of individuals to invest in forest management for risk reduction. Findlater et al. (2022) further support this by discussing the complexities of integrating diverse forest values, including health and ecosystem integrity, into climate-adaptive management decisions.

In our study, timber production as one of the main provisioning services (Appendix 1) is predominantly associated with status and traditional values, contrasting with recreation FES that align with health and self-fulfillment values. This dichotomy underscores a division where certain values highlight tangible benefits, such as economic security from timber production, while others underscore experiential and psychological benefits, such as personal well-being from recreational activities (Grammatikopoulou and Vačkářová, 2021; Jones et al., 2016). Recent studies further corroborate that forest recreation significantly contributes to enhancing physical health, improving mental well-being, and fostering self-fulfillment (Moseley et al., 2017; Riccioli et al., 2018). For instance, research by Lee et al. (2022) demonstrates a direct correlation between forest-based recreational activities and reduced stress levels, while Mantler and Logan (2015) emphasize the role of natural environments in promoting mental health and overall life satisfaction.

Hunting emerged as the least prioritized FES (Appendix 1), demonstrating substantial variability in responses. This variability suggests a divided perspective on the significance of hunting, likely influenced by demographic differences in attitudes toward this activity. The divergence in viewpoints underscores the necessity for adaptable management policies capable of accommodating a range of cultural perspectives. Flexible policy frameworks are crucial for balancing the ecological, social, and economic dimensions of forest ecosystem services, thereby ensuring sustainable management that respects diverse values and traditions. Also other studies highlight the complexity of managing hunting as an ecosystem service. For instance, Fischer et al. (2012) examined the socio-cultural dimensions of hunting across various regions, revealing significant differences in its perceived value. Our results support the findings in a study by Fagarazzi et al. (2021), who evaluated the economic value of hunting as a cultural ecosystem service. Their findings indicated that hunting has transitioned from a subsistence activity to an elitist recreational pursuit. We agree that the importance of forest ecosystem services, including hunting, varies significantly based on socio-cultural profiles. For example, regulating and cultural services are highly valued, whereas provisioning services like hunting are less prioritized by urban residents and those with higher education (Janeczko et al., 2023). Flexible and adaptable management policies are essential to address the diverse perceptions of hunting and ensure sustainable forest management. These policies must consider the ecological, social, and economic dimensions of forest ecosystem services (Garcia et al., 2018). We suggest that managing hunting as an ecosystem service requires flexible policies that accommodate diverse cultural perspectives and demographic differences. Such policies are crucial for balancing the ecological, social, and economic dimensions of forest ecosystem services, thereby ensuring sustainable management that respects diverse values and traditions.

## 4 Conclusion

The integration of basic human values with FES offers a pathway to more sustainable forest management. This study reveals that values such as health, tradition, and forest protection hold significant importance among the public, suggesting that forest management strategies must align with these core values to be effective and resonant. The notable variability in how different FES are prioritized by individuals underscores the necessity for flexible and adaptable policy frameworks. These frameworks should be inclusive of diverse values and interests, potentially through zoning strategies that designate specific areas for conservation, recreation, and sustainable hunting.

Aligning forest management practices with the public’s diverse values can foster a more inclusive and sustainable interaction with forest ecosystems. By acknowledging and incorporating the psychological dimensions of human-forest interactions into FES frameworks, this research advances our understanding of how human values shape forest management preferences. It paves the way for developing more effective, value-based approaches to sustainable forest management. Ultimately, this approach ensures that forest management is not only ecologically and economically viable but also culturally and socially relevant, respecting the diverse values and traditions of different demographic groups.

While our study focused on the content analysis and discriminant analysis, future research could benefit from incorporating additional methodologies such as prototypical analysis, similitude analysis, or the Reinert Method for Textual Data Clustering. These approaches could provide deeper insights into the complex relationships between human values and forest ecosystem services, offering a more nuanced understanding of the data. By applying these social representation methodologies, future studies could expand on our findings and explore the cognitive and emotional dimensions of forest-related values more comprehensively.

By further exploring these aspects, forest management practices can be enhanced to promote sustainability, ensuring that the psychological and cultural connections people have with forests are respected and integrated into management strategies. This holistic approach will contribute significantly to the national effort to maintain and enhance forest welfare services, fostering a deeper connection between individuals and forest environments.

## Data Availability

The raw data supporting the conclusions of this article will be made available by the authors, without undue reservation.
