# Supplementary material for: Integrating basic human values with forest ecosystem services: pathways to sustainable forest management
Source: Front Psychol. 2024 Oct 9;15:1444775. doi: 10.3389/fpsyg.2024.1444775 (PMC11496941; doi:10.3389/fpsyg.2024.1444775)
Supplement: Supplementary file 1 [file Table_1.DOCX]

**Appendix 1**

*Mean values and ± standard error (in parenthesis) of the five variables selected by the SLDA for basic categories of forest values. Values express the opinion of respondents about the importance of forest ecosystem services by 5-level Likert scale (1. not important at all. …. 5. highly important).*

| Basic categories of human values | Abbr. | Recreation (x_1_) | Timber  (x_2_) | Climate  (x_9_) | Hunting  (x_6_) | Protection  (x_4_) |
| --- | --- | --- | --- | --- | --- | --- |
| Sensual | sens | 4.07 (0.07) | 3.76 (0.08) | 4.72 (0.03) | 2.97 (0.08) | 4.70 (0.04) |
| Health | heal | 4.22 (0.06) | 3.93 (0.07) | 4.92 (0.03) | 2.85 (0.07) | 4.87 (0.03) |
| Safety | safe | 3.58 (0.11) | 4.33 (0.12) | 4.80 (0.05) | 3.03 (0.12) | 4.68 (0.05) |
| Status | stat | 3.67 (0.15) | 4.14 (0.17) | 4.76 (0.07) | 2.88 (0.17) | 4.69 (0.08) |
| Traditional | trad | 3.56 (0.11) | 4.17 (0.12) | 4.68 (0.05) | 3.26 (0.13) | 4.69 (0.06) |
| Relational | rela | 3.96 (0.05) | 3.73 (0.06) | 4.79 (0.03) | 2.77 (0.06) | 4.77 (0.03) |
| Aesthetic | aest | 4.09 (0.05) | 3.80 (0.06) | 4.82 (0.03) | 2.94 (0.06) | 4.85 (0.03) |
| Self-fulfillment | self | 4.20 (0.05) | 3.86 (0.05) | 4.82 (0.02) | 2.74 (0.05) | 4.78 (0.02) |
| Forest protection | prot | 3.78 (0.15) | 3.86 (0.17) | 4.92 (0.07) | 3.18 (0.17) | 4.76 (0.08) |
